# Domestic Correspondence

**Published:** 1889-04

**Authors:** 


### Domestic Correspondence.

To the Editor:

The " Hotel Gerlack " in New York City is a new and massive building with the most modern and elaborate fittings and appointments.

On the evening of March 11th, in the magnificent dining-room of this fine hotel, were arranged long rows of tables furnished and decorated in a sumptuous and artistic manner. It was the occasion of a complimentary dinner given by the dentists of New York, Brooklyn and New Jersey to their esteemed friend, Dr. Wm. H. Atkinson. In every chair at the tables was seated a happy occupant, each displaying a floral decoration.

During the early part of the evening a number of ladies, guests of the hotel, appeared at the doors of the banquet hall to witness the large gathering of joyous gentlemen who had assembled at the " gay and festive " board. Dr. C. E. Francis presided on the occasion. At his right appeared the honored guest of the evening, and at his left sat the much respected Dr. Wm. H. Dwinelle, to whom was tendered a similar banquet a year ago, on the completion of his fiftieth year of dental practice.

After all were seated, Rev. Dr. Bachus, of New York, at the request of the President, asked Divine blessing. Next came the army of waiters, well trained, polite and mannerly, and the good things were provided for the " new comfort " were profusely served.

At the conclusion of the feast the President called to order the mirthful assembly, and in a brief speech paid a worthy tribute to the honored guest, " whose name," said he, " is as familiar as a household word to every intelligent dentist in the enlightened portion of the world, and whose labors for the benefit of his fellow practitioners and the whole specialty has not been equaled by that of any other individual."

The following " toasts " presented by the committee were then read by the President:

" Dr. Atkinson, our guest."—" His eminent attainments have won our admiration. His devotion to the welfare of the profession he loves and adorns, and his unceasing efforts for its increased efficiency, not only in this city of his residence, but elsewhere as well, wil. make his name to be ever gratefully remembered; and the recollection of his acquaintance and friendship a perennial cause of gladness to every New York dentist who has been associated with him during these years of active life."

Dr. Wm. Dwinelle being called upon to respond to this sentiment, paid many just compliments to Dr. Atkinson, and referred to his life of action, work, and useful labors among his professional brethren. The President then read his second toast. "Dr. Atkinson, our guest."—"As a man and a citizen, we respect him. As a professional brother, we highly esteem him. As a tireless leader in scientific research, a fearless propounder of the truth discovered, a generous giver of the fruits of his patient toil in this field, we honor him. As a warm friend and willing helper of everyone who is honestly struggling along the same road toward light and improvement, we love him." The President called upon Dr. C. A. Marvin, of Brooklyn, to respond, and the doctor proved equal to the occasion. His graceful speech and glowing peroration called forth showers of applause.

The third toast was then presented :

"Dr. Atkinson, our friend."—"Ever-ready to lend a helping hand to the earnest but weary student; to hold out a light for stumbling feet; to impart his wisdom to them that lack; to add inspiration by his stirring words to the discouraged; to stimulate by his unconquerable ardor the search for truth when it is in danger of flagging, and the effort for professional progress when it halts; his name will be remembered, honored and loved by his professional brethren in every city and in every land whither they may direct their steps."

Dr. C. S. Stockton, of New Jersey, spoke in response to this toast. He praised the generous deeds of Dr. Atkinson, whom he pictured as a true and valuable friend to the members of his profession.

Rev. Dr. Backus was then introduced, who made a brief congratulatory speech; after which the President requested all present to rise and unite with him in drinking to the health of their "citizen, guest and friend," and wishing him many years of happiness and prosperity. This was done with a hearty good will.

The President then introduced the guest of the evening, whom he requested to speak in his own defence.

Dr. Atkinson's speech was followed by a few appropriate remarks from Dr. Heitzman, and the happy gathering dispersed.

Much credit is due to Drs. Northrop, Lord and Hill, of the committee, for the excellent arrangements for the dinner.

X.

To the Editor:—A dinner was given by members of this Society to their out-of-town guests on Tuesday afternoon, March 19th, at Hotel Brunswick, Fifth Avenue, New York. Among those present were Dr. H. C. Merriam, of Salem, Mass.; Drs. Niles, Hopkins, and Potter, of Boston; Dr. Geary, of Lowell; Dr. L. S. Straw, of Newburgh; Dr. C. N. Peirce, and W. X. Sudduth, of Philadelphia; Dr. Geo. Parmly, of Hartford; Dr. Geo. Mosley, of Buffalo; Dr. J. Palmer, of New Brunswick; Dr. S. G. Palmer, of Syracuse; and Dr. C. S. Wardell, of Stamford, Ct. Many prominent dentists of New York and Brooklyn were also at the table. Social topics were discussed in a pleasant conversational way. After dinner was over the entire party wended their way to the parlors of the Academy of Medicine to attend the regular monthly meeting of the Odontological Society, a brief report of which appeared in the New York *Tribune*, as follows:

"The Odontological Society of New York met last night at the Academy of Medicine, No. 12 West Thirty-first Street, to listen to a paper by Dr. H. C. Meriram, of the Harvard Dental School, on 'Professional Atmosphere and Morals.' He would class dentistry as a 'specialty in medicine,' and maintained that the only reason why, with most of its practitioners, it had not been raised above the level of a trade, was that dentists had encouraged and supported methods which tended to make their profession decidedly illiberal. The chief difficulty, he said, was that some members of the profession had invented devices, appliances, materials, instruments, etc., which they had patented and sold to a combination which oppressed the whole fraternity by charging exhorbitant prices and imimposing annoying conditions for the use of the articles so controlled."

"He pointed out the fact that no such condition existed in the liberal professions, the lawyer, minister and physician having free access to all that his brothers achieved, and hoped the day would come when the same could be said of dentistry. A discussion followed the reading of the paper." X.

---

To the Editor:

The annual meeting of the Louisiana State Dental Society was held in New Orleans, March 5th and 6th, 1889, the President, Dr. C. B. Johnson (Monroe), in the chair.

The Executive Committee had quite recently decided to postpone the meeting to a later date, but, owing to some misunder standing, notice of the intended change was not issued in time to reach a number of members in distant parts of the state, who arrived in the city, expecting the usual meeting on the date appointed.

A rather informal meeting was therefore held, the President's annual address being the only paper read.

A resolution was adopted inviting the Mississippi State Dental Society to hold a joint meeting with the Louisiana State Society.

Dr. Bauer announced the reception of a letter from the Secretary of the International Dental Congress, asking the appointment of a delegate to attend the meeting of that body in Paris, September 1st, 1889.

The election of officers was held, with the following result: President, Dr. Jno. W. Adams; 1st Vice-Pres., Dr. Massingill; 2d Vice-Pres., Dr. Comeygs; Rec. Sec., Dr. J. G. McCodloch; Cor. Sec., Dr. C. E. Kells, Sr.; Treas., Dr. P. J. Friederichs. Board of Dental Examiners: Dr. C. B. Johnson, G. J. Friederichs, J. Bauer, Chas. Eckardt, M. Viet. Executive Committee: Drs. A. G. Friedrichs; I. J. Savrazin, Chas. Eckardt, E. Telle, J. G. McCulloch, A. J. Friedrichs.

The Society then adjourned to Wednesday, February 19th, 1889. MRS. J. M. W.

---

TO THE EDITOR:—In answer to your letter of inquiry regarding my engine and mallet I claim the following novel features: The power can be applied either by foot or motor, when by the latter it is converted into a compensating suspension engine. The radius of the arm can be positively held at any desirable point in either engine. The arm of the foot engine is connected at the shoulder at top of pedestal, by an universal joint, which is capable of being thrown, and used with the same results on either side of the pedestal, making it right and left handed without drawing the cord across the pedestal or standard. The idlers throughout are mounted on a central pivot, which permits the cord running over them to draw automatically to the greatest tension, and thereby equalizing the strain in any and every position that the arms are placed, allowing it to run as freely with the arms folded to their extreme point, as when lengthened to their full extent. In other words, by a crucial test which was made upon the engine by a wire passing around the driving wheel, and the arm held in a perfectly vertical position, and the wire following its course, and drawn tightly, allowed the arms to be folded to their minimum length, without any apparent tension upon the wire. This has never been accomplished in any other engine. The idlers of the elbow and wrist joint carry out the same device as those of the shoulder, which gives the engine great power. When not in use as slip joint at the shoulder, allow the arm to be dropped at the side of the standard—out of the way.

The Mechanical Mallet contains several novel features, among which is an impact anti-friction roller on the periphery of a wheel that is hinged above, and comes in contact with the head of the spindle. It is adjustable, to be driven by a cord, or by the axle caught in a hand-piece, making the mallet adaptable to all kinds of engines in use. As the wheel is rotated, the impact anti-friction roller coming in contact with the head of the spindle, rolls over it, producing pressure akin to the action of the hand and wrist, being a mechanical imitation of the push blow, as an instrument would be used in the hand. The struck end of the spindle may be made either solid from end to end, or an additional anti-friction roller may be placed in the contact end, making two anti-friction rollers, relieving each other; or a rubber plunger may be used to receive the blow and transmit it to the spindle. These devices lesson the noise, and the push movement of the instrument, over the hammer blow, either of spring or cam movement, is very much more tolerant to the patient, and effective in its working properties. The regulating device for increasing and diminishing the blow is very simple and effective, being arranged in either of two ways. First, by a swivel joint catching a capstan threaded below it which cannot revolve, and a series of notches in a circle on the outer surface of the swivel which receive a spring, and are capable of being turned to the right or left, as it is desirable to increase or decrease the stroke. The second device differs from this only in that the swivel is unbroken, working directly upon a screw movement. Below the swivel movement is a second swivel which allows the instrument with point in place to be revolved in the hand. It is finished in vulcanized hard rubber, which makes a very pleasant contact with the fingers. The receptacle for holding the plugging instruments is made in two forms, one to receive the Snow and Lewis bit points, and the other to receive the electrical plugger points. The entire engine and mallet is simple throughout in its construction, capable of doing effective work, with less liability of getting out of order than any other engine or mechanical mallet of which we know.—*H. C. Register.*

---

To the Editor:

Ten Dont's for your readers:—

1. Don't in any way annoy your patient if you can possibly avoid it. By studying his comfort while he is with you, you will get dollars for it later on.

2. Don't pick around a cavity, or make any unnecessary scratching; it is very irritating, and often more hard to bear than a good clean cut that tells.

3. Don't use thick rubber dam. It acts like rubber wedges, and makes it necessary to crowd the gum back further in order to get beyond the cervical border of the cavity, often causing great pain.

4. Don't use large ligatures for tying on the rubber. It crowds the gum instead of slipping under its free margin. Common white sewing silk, "C," well waxed, I use in nearly every case. D. M. C.

---

To the EDITOR:

The use of mer. bichlor. is now so common that any means that can be employed to disguise its very unpleasant taste should be hailed with satisfaction. With some patients the taste is most disagreeable and persistent.

For some time I have been using rose-water as a dilutent, and find it works well. I keep a one per cent. bichlor. solution ready prepared, and mix it with the rose-water when wanted—generally in the proportion of one part of the former to nine of the latter. Patients notice the change in taste of the "bug poison," and speak of it very pleasantly. GEO. S. ALLAN.

---

To the EDITOR:

Miss Emma Prewitt, honorary member and "adopted daughter" of the Southern Dental Association—daughter of the lamented Dr. J. H. Prewitt, Madisonville, Ky.—is probably the youngest authoress in the United States. When but a mere child she gave evidence of unusual descriptive powers, which were first displayed in contributions to the local daily papers, and also in several magazine articles published before she was thirteen years old.

Though but sixteen years old at present, Lippincott & Co. have published the novel, entitled KARLINE HOY, to which many of the dentists present at the meeting last summer at Louisville, subscribed.

Her future is rich in promise, both in literature and music, and its unfolding will be watched with interest by every member of that profession which calls her "daughter" in honor of the father who so suddenly passed away in the prime of manhood, but little more than a year ago.

We began the perusal of Miss Prewitt's book on account of the authors relation to the profession and finished it with increasing interest on its own accounts. DENTOS.

